# Traumatic brain injury, working memory-related neural processing, and alcohol experimentation behaviors in youth from the ABCD cohort

**DOI:** 10.1016/j.dcn.2024.101344

**Published:** 2024-01-18

**Authors:** Everett L. Delfel, Laika Aguinaldo, Kelly Correa, Kelly E. Courtney, Jeffrey E. Max, Susan F. Tapert, Joanna Jacobus

**Affiliations:** a SDSU / UC San Diego Joint Doctoral Program in Clinical Psychology, USA; b University of California, San Diego, Department of Psychiatry, USA

**Keywords:** TBI, Development, Cognition, Alcohol, Neuroimaging, FMRI

## Abstract

Adolescent traumatic brain injury (TBI) has long-term effects on brain functioning and behavior, impacting neural activity under cognitive load, especially in the reward network. Adolescent TBI is also linked to risk-taking behaviors including alcohol misuse. It remains unclear how TBI and neural functioning interact to predict alcohol experimentation during adolescence. Using Adolescent Brain Cognitive Development (ABCD) study data, this project examined if TBI at ages 9–10 predicts increased odds of alcohol sipping at ages 11–13 and if this association is moderated by neural activity during the Emotional EN-Back working memory task at ages 11–13. Logistic regression analyses showed that neural activity in regions of the fronto-basal ganglia network predicted increased odds of sipping alcohol by ages 11–13 (*p* < .05). TBI and left frontal pole activity interacted to predict alcohol sipping (OR = 0.507, 95% CI [0.303 - 0.846], *p* = .009) – increased activity predicted decreased odds of alcohol sipping for those with a TBI (OR = 0.516, 95% CI [0.314 - 0.850], *p* = .009), but not for those without (OR = 0.971, 95% CI [0.931 −1.012], *p* = .159). These findings suggest that for youth with a TBI, increased BOLD activity in the frontal pole, underlying working memory, may be uniquely protective against the early initiation of alcohol experimentation. Future work will examine TBI and alcohol misuse in the ABCD cohort across more time points and the impact of personality traits such as impulsivity on these associations.

Adolescence is a time of both physical and psychological development where youth experience greater autonomy that can also increase risk for reckless behaviors and subsequent injury (Crone and van Duijvenvoorde, 2021). This is theorized to stem from pubertal brain changes associated with increased reward seeking alongside ongoing development in frontal brain regions related to these behaviors (Steinberg, 2008). One common, yet not-well understood phenomenon is traumatic brain injury (TBI), which is an injury to the head or neck that can disrupt normative brain functioning (Centers for Disease Control and Prevention, 2019). These injuries are very common amongst adolescents due to a wide range of factors (e.g., falls, sports injuries) with as many as 3.5 million total reports of TBIs in a given year (e.g., 2009; Coronado et al., 2012). Epidemiology research conducted in the United States over a six-year period found that for children younger than 15 years of age, that yearly there were approximately 2685 TBI-related deaths, 37,000 hospitalizations, and 435,000 emergency department visits; children ages 0–4 accounted for the largest quantity of TBI events and TBI-related hospital visits compared to children ages 5–9 and 10–14 (Langlois et al., 2006). Notably, estimates from a large British Columbian youth sample (40,000), suggest approximately 12% of adolescents have experienced a TBI and 4% have had 2 or more TBIs in the past year (mean age 14.9; Rackley et al., 2017). As such, adolescents and young adults experience some of the highest rates of TBI-related emergency room visits in North America. For example, in 2017, amongst children ages 0–17, TBIs accounted for approximately 7.8% of all TBI-related hospitalizations and approximately 4.5% of all TBI-related deaths (Centers for Disease Control and Prevention, 2021).

TBIs in individuals younger than age 15 are related to a wide range of negative outcomes, such as school and behavioral problems (Hawley et al., 2004), mental health symptoms (e.g., ADHD; Max et al., 2005), and alcohol misuse (Bogner et al., 2019). Alcohol use is especially important to examine in adolescence as the majority of high school aged adolescents steadily increase their alcohol use throughout high school (Bray et al., 2022), ranging from 23% having used alcohol in 8th grade to 61% in 12th grade (Miech et al., 2023). Although the vast majority of adolescents experiment with alcohol, earlier age of experimentation with alcohol (< 15 years of ag) is associated with a range of negative outcomes such as poorer neuropsychological and emotional functioning in early adulthood (Nguyen-Louie et al., 2017), depressive feelings that increase as alcohol use increases (Kleinjan et al., 2015), structural brain changes in frontal brain cortices (Infante et al., 2018), and the development of alcohol-related disorders over the lifespan (DeWit et al., 2000). Given the range of negative outcomes associated with alcohol experimentation during childhood and early adolescence (∼ages 10–14), it is necessary to examine this unique developmental window as an important period to deploy prevention approaches, and also identify risk factors that often co-occur with early life alcohol and substance misuse. Research shows that early life TBI is related to alcohol misuse; although this relationship may be accounted for by personality characteristics common to those who experience TBI and engage in early-life substance use, such as impulsivity which has been associated with alcohol experimentation in youth as young as age 9 (Ilie et al., 2013; Watts et al., 2021). While it remains unclear about what pre- and/or post-injury TBI-related factors may contribute to adolescents' alcohol experimentation, it is possible that alterations in brain functioning may explain some outcome differences.

Functional magnetic resonance imaging (fMRI) can be used to identify subtle neurological alterations in brain functioning following mild TBI that behavioral assessments are unable to detect (Mayer et al., 2011, Yang et al., 2012). Working memory is one aspect of inhibitory control that is of particular interest with regards to TBI. A systematic review of TBI and working memory (school-aged children and adolescents under 20) indicated that children who experience TBI are at risk of demonstrating subsequent working memory deficits (Phillips et al., 2017). More severe injuries and younger age of injury (i.e., prior to age 8) were also associated with increased working memory deficits. This review demonstrated the unique vulnerability that TBIs may confer upon working memory outcomes during childhood.

One major brain system of interest that is related to both TBIs and alcohol use is the fronto-basal ganglia network, which has a major role in inhibitory control (Aron et al., 2007). TBIs of greater severity (moderate to severe TBI) are associated with increased blood-oxygen-level-dependent (BOLD) activity in the frontal cortex (in participants ranging from adolescence to older adulthood during an adaptive task; Olsen et al., 2015) and in the fronto-basal ganglia network (in young adult males; Hermans et al., 2017) during working memory and cognitive control tasks (i.e., Not-X go/no-go type task and a Stop-Signal task). Similarly, Huang et al. (2023) found that increased gamma activity in the frontal pole, using magnetoencephalography at rest, was associated with poorer post-concussion outcomes in adolescents. Overall, it appears that increased brain activity has been identified post injury during both cognitive tasks (Hermans et al., 2017, Olsen et al., 2015) and at rest (Huang et al., 2023). Decreased default mode network connectivity, particularly within frontal regions, during rest in a sample of young adults may relate to increased fatigue and cognitive deficits post TBI (Mayer et al., 2011). After initiating heavy alcohol use, adolescents followed from ages 14 to 18 show decreased BOLD activity when trying to inhibit responses in a go/no-go task in areas of the frontal lobe and putamen compared to non-drinkers Wetherill et al. (2013)). Yet, it is still unclear how regions of the fronto-basal ganglia network function under a cognitive load in adolescents with a TBI and how this might predict later alcohol use and alcohol-use related behaviors.

The Adolescent Brain Cognitive Development (ABCD) study is a longitudinal study of 9 and 10-year-old participants sampled from 21 United States-based sites across 10 years (Volkow et al., 2018). TBI and its association with alcohol use has not been extensively examined in the ABCD study to date. An initial descriptive study in ABCD identified approximately 4% of participants who have had a TBI (Dufour et al., 2020), where a TBI was operationalized as a loss of consciousness, short-term amnesia, or an altered memory state. This study also identified that being male, higher socioeconomic status, somatic symptoms, and ADHD symptoms (prior to multiple comparison correction) were correlated with TBI in the ABCD cohort. A second ABCD cohort study examined the association between sports participation with head and neck injuries. Results indicated that 0.8% had a TBI with loss of consciousness, where participation in contact sports and longer duration of sports participation was associated with head and neck injuries (Veliz et al., 2021). Additionally, TBIs (with or without loss of consciousness) are associated with a wide range of psychological symptoms (i.e., internalizing, externalizing, and overall psychological symptoms), sleep disturbances (Sheth et al., 2022), and gender differences demonstrating that male participants had more TBIs than female participants in ABCD (Veliz and Berryhill, 2023). Only one paper to date has examined neuroimaging indices among those with TBI in the ABCD study, examining mild TBI, including those with a TBI without a loss of consciousness, or with a loss of consciousness less than 30 min (Lopez et al., 2022). This paper found that TBIs were associated with poor mental health outcomes in the ABCD study across Baseline (mean age 9.92), Year 1 (mean age 10.92), and Year 2 (mean age 12.00), alongside examining the mediating effect of brain volume (e.g., total cortical volume, cortical thickness, white matter volume, ventricle volume, gray/white matter contrasts) on the association between TBI and various mental health factors (e.g., distress scores and total mental health problem scores). These mediation analyses were however non-significant, but it was found that TBI was related to lower levels of cerebrospinal fluid volume overall.

Although much research has shown associations between adolescent TBI and negative behavioral outcomes (e.g., Bogner et al., 2019; Hawley, 2004; Max, 2014), the associations between lifetime mild and moderate TBI on neural activity in the fronto-basal ganglia network and alcohol use has yet to be examined. Given that many of the previous studies on adolescent TBI in the ABCD cohort have focused on the impact of TBI without a loss of consciousness, we sought to examine the impact that more severe TBIs, with a loss of consciousness, has on neural functioning and sipping behaviors. Therefore, this study has 3 primary research aims: (1) to examine if presence of TBI at ages 9–10 independently predict increased odds of alcohol sipping at ages 11–13, (2) to examine if increased BOLD activity in the fronto-basal ganglia network during the Emotional N-Back task (EN-Back) at ages 11–13 independently predicts increased odds of alcohol sipping at ages 11–13, and (3) examine if TBI and BOLD activity in the fronto-basal ganglia network will interact whereby increased BOLD activity will be more strongly associated with increased odds of alcohol experimentation for participants with a TBI as compared to those not reporting a TBI by ages 9–10 in the ABCD cohort (Wetherill et al., 2013; Olsen et al., 2014; Watts et al., 2021). All of these planned analyses will account for the necessary covariates that may be exerting a systematic effect on the moderating effect of BOLD activity in the fronto-basal ganglia network and the association between TBI and alcohol experimentation, such as demographic characteristics and trait impulsivity.

## 1 Materials and methods

This study used longitudinal baseline and year 2 follow-up data from the ABCD Data Release 4.0. This study has a total of 11,876 participants and is funded by the National Institutes of Health (Volkow et al., 2018). The University of California, San Diego Institutional Review Board approved all aspects of this study for the ABCD consortium. Parental consent and adolescent assent was obtained prior to participating in the study.

### 1.1 Recruitment

The ABCD study primarily recruited from schools local to each study site using a probability sample, where school selection is determined by sex, race and ethnicity, socioeconomic status, and urbanicity. Interested youth and their families first completed a brief eligibility interview over the phone to confirm they were between age 9–10 and have no MRI contraindications (e.g., a metal implant or pacemaker; Garavan et al., 2018).

### 1.2 Procedure

Youth participants and their parent/guardian attended study sessions at their local research site for their baseline (ages 9–10) and follow-up visit (ages 11–13) approximately 2-years later. Visits were completed separately for youth and their parent/guardian to maintain confidentiality. Both baseline and year 2 follow-up visits included questionnaires, neurocognitive testing, biological samples and an MRI scan (Lisdahl et al., 2018, Luciana et al., 2018, Uban et al., 2018). Study sessions at each visit were completed throughout an 8-hour research session or two 4-hour sessions. Parents and youth were compensated financially and with other prizes given for their participation.

### 1.3 Demographic information

Demographic information was collected from parent self-report on the youth participant’s biological sex and ethnicity. Biological sex was then recoded into a dichotomous variable (i.e., female or male). For race/ethnicity, parents reported if their child is White (1), Black (2), Hispanic (3), Asian (4), or Other (5), which was used for subsequent descriptive analyses. Total combined household income was recoded into a dichotomous variable (i.e., 99k or less, or greater than 100k) and combined parental education was also recoded into a dichotomous variable (i.e., high school graduate or less, or education beyond high school) for preliminary analyses (see Table 1 and 2). When entered as a covariate, it was entered as a continuous variable ranging from income brackets of 1 through 10 (1 = <$5000; 2 = $5000–12,000; 3 = $12,000–16,000; 4 = $16,000–25,000; 5 = $25,000–35,000; 6 = $35,000–50,000; 7 = $50,000–75,000; 8 = $75,000–100,000; 9 = $100,000–200,000; 10 = >$200,000]. Internalizing and externalizing T-scores were also derived from the Child Behavior Checklist (CBCL; Achenbach, 1999).

**Table 1:** Demographic and behavioral characteristics at baseline.

	**TBI-** **98.87%** **(11,742/** **11,876)/** **M (SD)**	**TBI+** **0.01%** **(134/** **11,876)/** **M (SD)**	**Chi**^**2**^***p*****-value**
Race/Ethnicity (% White) (% Black) (% Hispanic) (% Asian) (% Other)	52.0% 6102/ 11,742 15.1% 1775/ 11,742 20.3% 2382/ 11,742 2.1% 252/ 11,742 10.5% 1231/ 11,742	57.8% 78/ 134 7.4% 10/ 134 22% 30/ 134 0% 0/ 134 11.9% 16/ 134	*p* = .048 *
Biological Sex (%Male)	52.1% 6117/ 11,742	59.0% 79/ 134	*p* = .135
Household Income (% <99 K/year)	59.6% 6998/ 11,742	53.0% 71/ 134	*p* = .144
Highest Parental Education (% HS Graduate or below)	13.1% 1463/ 11,310	8.5% 11/ 130	*p* = .092
			***T*****-Test** ***p*****-value**
Internalizing T-Score	48.4 (10.61)	52.9 (12.14)	*p* < .001 *
Externalizing T-Score	45.7 (10.30)	49.5 (12.37)	*p* < .001 *
Age (In Months)	119.0 (7.49)	119.2 (8.01)	*p* = .764
EN-Back Task Accuracy (% correct, 2-Back Condition)	85.6% (7.74)	86.3% (0.11)	*p* = .569
UPPS-P Negative Urgency	8.49 (2.64)	8.83 (2.75)	*p* = .200
UPPS-P Positive Urgency	7.99 (2.96)	8.55 (3.17)	*p* = .040 *
UPPS-P Lack of Planning	7.74 (2.38)	7.77 (2.27)	*p* = .900
UPPS-P Sensation Seeking	9.77 (2.68)	9.98 (2.64)	*p* = .400
UPPS-P Lack of Perseverance	7.04 (2.25)	6.97 (2.64)	*p* = .800
Alcohol Sipping	10,576 (No sip)	116 (No sip)	*p* = .200
Alcohol Sipping	1166 (Yes Sip)	18 (Yes Sip)	*p* = .200
EN-Back Task Accuracy (% correct, 2-Back Condition)	85.4%	87.5%	*p* < .001

Chi-square goodness of fit tests identified potential demographic differences by TBI-status at baseline and alcohol sipping at 2-year follow-up. Analyses indicated that there was a significant difference in TBI-status by race/ethnicity (χ^2^ = 9.59, *p* = .048). White participants accounted for the highest rates of TBIs (57.8%), followed by Hispanic participants (22%), participants identifying as “Other” (11.9%), Black participants (7.4%), and Asian participants (0.0%; see Table 1 for a full breakdown of TBI by race/ethnicity statistics). There was no sex (χ^2^ = 2.23, *p* = .135), household income (χ^2^ = 2, *p* = .100), or parental education (χ^2^ = 2.84, *p* = .092; see Table 1). There was a significant difference between internalizing and externalizing T-scores, where there were higher scores in the TBI-positive group (*p* < .001).

There was a significant difference in alcohol sipping by race/ethnicity (χ^2^ = 171.23, *p* < .001). White participants accounted for the highest rates of sipping (68.5%), followed by Hispanic participants (13.6%), participants identifying as “Other” (10.2%), Black participants (5.9%), and Asian participants (1.8%; see Table 2 for a full breakdown of sipping by race/ethnicity statistics). The alcohol sipping group (*p* < .001) had higher internalizing and externalizing T-scores and were older as compared to the non-sipping group (*p* < .001). Additionally, there was a significant difference in alcohol sipping by sex (χ^2^ = 7.205, *p* = .007), with males reporting greater alcohol sipping than females, and sex was included in all models as a covariate. Household income also differed by alcohol sipping (χ^2^ = 109, *p* < .001), with higher income individuals reporting greater alcohol sipping than lower income individuals. Similarly, parental education differed by alcohol sipping (χ^2^ = 51.66, *p* < .001), as individuals with higher parental education reported more alcohol sipping than individuals with lower parental education. Although sex, race/ethnicity, parental education, and household income differed by alcohol sipping, sex and household income were selected as covariates given that sex (Tomasi and Volkow, 2023) and socioeconomic disparities have been associated with structural brain differences in the ABCD cohort; sensitivity analyses in the cohort have demonstrated similar results regardless if race and ethnicity factors are controlled for, as conducted by Weissman et al. (2023). Similarly, Dumornay et al. (2023) found that seemingly race-related brain differences were related to facets of adversity such as economic hardship in the ABCD cohort.

**Table 2:** Demographic and behavioral characteristics at baseline.

	**ALC- 90.03%** **10,692/** **11,876** **M (SD)**	**ALC+ 11.00% (1184/** **11,876)** **M (SD)**	**Chi**^**2**^***p*****-value**
Race/Ethnicity (% White) (% Black) (% Hispanic) (% Asian) (% Other)	50.2% 5369/ 10,692 16.0% 1715/ 10,692 21.1% 2251/ 10,692 2.2% 231/ 10,692 10.5% 1126/ 10,692	68.5% 811/ 1184 5.9% 70/ 1184 13.6% 161/ 1184 1.8% 21/ 1184 10.2% 121/ 1184	*p* < .001 *
Biological Sex (%Male)	51.8% 5534/ 10,692	55.9% 662/ 1184	*p* = .007 *
Household Income (% <99 K/year)	61.1% 6532/ 10,692	45.4% 537/ 1184	*p* < .001 *
Highest Parental Education (% HS Graduate or below)	13.8% 1401/ 10,278	6.2% 73/ 1184	*p* < .001 *
			***T*****-Test** ***p*****-value**
Internalizing T-Score	48.4 (10.62)	49.2 (10.77)	*p* = .010 *
Externalizing T-Score	45.6 (10.32)	46.7 (10.43)	*p* < .001 *
Age (In Months)	118.9 (7.46)	119.9 (7.74)	*p* < .001 *
TBI	10,576 (No TBI)	1166 (No TBI)	*p* = .200
TBI	116 (Yes TBI)	18 (Yes TBI)	*p* = .200

### 1.4 Traumatic brain injuries baseline assessment

Participants completed the Ohio State University TBI Identification Method (Bogner et al., 2017) at baseline to assess lifetime history of TBI. Screening questions were asked to identify any injuries to the head or neck that may have resulted in a TBI, with follow-up questions about any post-injury symptoms. Lifetime TBI was the focus of this study and reported TBIs could have occurred anytime from birth up until study date. Using the Ohio State University TBI Identification Method, parents were interviewed about their child and reported on history of any head injury and any head injury treatment in an emergency room, as well as any history of experiencing a TBI with a loss of consciousness. This variable was then recoded into a dichotomous variable reflecting either no history of TBI or mild-severe TBI (i.e., history of TBI with no loss of consciousness or has had at least one TBI with loss of consciousness). A head or neck injury was only coded as a TBI if there was a loss of consciousness to only include injuries that induced an altered cognitive state. Approximately 96.2% of the sample reported no or an improbable TBI (*n* = 11,419), 2.71% reported a possible mild TBI (*n* = 322), and 0.01% of the sample (*n* = 135) reported a TBI with loss of consciousness at baseline.

### 1.5 Impulsivity baseline assessment

The UPPS-P was administered at baseline to examine five subscales of impulsivity (i.e., Negative **U**rgency, **P**ositive Urgency, Lack of **P**lanning, **S**ensation Seeking, and Lack of **P**erseverance; Barch et al., 2018). The version of the UPPS-P used in ABCD is a short-form measure of 20 items where four items correspond to each subscale. Participants responded to each item from 1 (not at all like me) to 4 (very much like me) for normally coded items (e.g., “I like new, thrilling things, even if they are a little scary), and from 1 (very much like me) to 4 (not like me at all) for other items (e.g., ”I am very careful”). The UPPS-P has demonstrated invariance and external invariance in the ABCD study (Watts et al., 2020) and was examined in the context of this study as a potential covariate. Analyses were conducted to examine potential differences in each of the five UPPS-P subscales by TBI-status. Only the Positive Urgency subscale differed by TBI-status (*p* = .040), which was entered as a continuous covariate into the model to control for impulsivity traits.

### 1.6 fMRI EN-back task year 2 assessment

Complete information of the EN-Back Task is described by Casey et al. (2018). The EN-Back Task was administered at Year 2 and examines working memory and emotional processing across a range of regions of interest (ROIs) implicated in online monitoring, remembering, and manipulating information as well as emotional reactivity (e.g., dorsolateral prefrontal cortex, hippocampus). This paradigm has been found to be an effective method of producing a working memory load and subsequent responses in ROIs related to working memory, especially in areas of the frontal and parietal regions (Owen et al., 2005). Participants were presented with an image of a sad, happy, or neutral face or a place and told to respond if it was a “Match” or a “No Match” to a previously presented image. There is a 0-back condition, where participants respond to each image by pressing a button if it matches the first image that was presented and there is a 2-back condition, where participants respond to each image if it matches the image presented two trials back. The task included one run of the 0-back with 8 blocks and one run of the 2-back with 8 blocks. Each block included 10 trials where each image was presented for 2.5 s for a total of 160 trials. There was not a significant difference in behavioral performance on the EN-Back (i.e., correct responses) based on TBI-status (*p* = .569). There was a significant difference in behavioral performance based on alcohol sipping-status, where those who have sipped (87.5%) performed significantly better than those who have not sipped (85.4%; *p* < .001).

The EN-Back Task mean beta weights from all ROIs representing the main contrast 2-back vs. 0-back across all emotional stimulus types were used for analyses. A total of 20 ROIs (10 per hemisphere) from the baseline EN-Back Task were examined, focused on areas of the fronto-basal ganglia network including: caudate, putamen, pallidum, accumbens, caudal middle frontal, lateral orbito frontal, medial orbito frontal, rostral middle frontal, superior frontal, and frontal pole.

### 1.7 Alcohol experimentation behavior 2-year follow-up assessment

A complete description of the ABCD environmental, health, and mental health questionnaires are described elsewhere (Barch et al., 2018, Zucker et al., 2018, Lisdahl et al., 2018). As part of the ABCD Youth Substance Use Introduction and Patterns questionnaire at 2-year follow-up, participants were asked how many times they have had a sip of alcohol. This variable was then recoded into a dichotomous variable (i.e., has not sipped alcohol or has sipped alcohol) as only 26 participants had more than 50 sips (range 0–385). Approximately 11% of the sample (*n* = 1184) reported sipping at 2-year follow-up.

### 1.8 Imaging data acquisition and processing

Specific details of the ABCD MRI and fMRI acquisition and scanning parameters are described elsewhere (Casey et al., 2018). After completion of each scan, scan data were uploaded to a shared server and processed accordingly by the Data Analytics and Information Core (DAIC) of the ABCD (Hagler et al., 2019). Scan parcellations were based on the Desikan-Killiany Atlas focusing on 68 cortical and 30 subcortical regions. Only EN-Back Task scans that passed the ABCD Data Analysis, Informatics, and Resource Center (DAIC) quality checks were included in the analyses for all 10 bilateral ROIs.

### 1.9 Missing data

Multiple imputation with chained equations were conducted using the Multivariate Imputation by Chained Equations (MICE) package (Van Buuren and Groothuis-Oudshoorn, 2011) in R Studio. This was conducted to help minimize systematic bias related to missing data through imputing all missing variables used in analyses except for race, sex, and study site, ending with a final sample size of 11,876. Although there was no significant difference in missing data between the sipping groups (χ^2^ = 0.02, *p* = .900) and the TBI groups (χ^2^ = 0.10, *p* = .700), there was a significant difference in missing data based on highest household income, where those with missing data had lower household income (6.88) than those with higher household income (7.27; *t* = 8.00, *p* < .001). The MICE package was used to produce a total of 50 new datasets using the observed data (Rubin, 1987), where one dataset was selected that was very similar to the original preimputation dataset. Previous studies have used a similar approach to accounting for missing data in the ABCD study (e.g., Aguinaldo et al., 2022; Roffman et al., 2021).

### 1.10 Data analysis

Preliminary analyses were conducted using Chi-square goodness of fit tests and t-tests to identify potential covariates. Using the Generalized Estimating Equation (GEE) Package (Geepack; Halekoh et al., 2006; Yan and Fine, 2004; Yan, 2002) in R Studio version 4.1.2 alongside ABCD Data Release 4.0., a series of multilevel moderated logistic regression GEE models were conducted to determine if the presence of a TBI at baseline predicts the odds of alcohol experimentation at two-year follow-up, and if this relationship is moderated by BOLD activity during the EN-Back Task at two-year follow-up in 10 a priori bilateral ROIs throughout the fronto-basal ganglia network. All analyses were nested by ABCD study site (i.e., 21 research study sites) and by family ID (which accounts for siblings that both participated in the ABCD study), alongside controlling for household income, biological sex, and UPPS-P Positive Urgency.

An exchangeable working correlation structure was utilized in the Gee models that assumes observations within each cluster of data (i.e., study site) are correlated together. This model helps to account for the unique correlations between participants in the same site. Although the statistical model accounts for a within-subjects factor (i.e., time), only the between-subject factors (i.e., TBI, BOLD activity, and alcohol experimentation) will be interpreted as time was not directly measured, but built into the statistical model design where TBI and BOLD activity were analyzed at baseline and alcohol experimentation at year 2 follow-up.

## 2 Results

### 2.1 Primary analyses

#### 2.1.1 Main effects

**Fig. 1:**
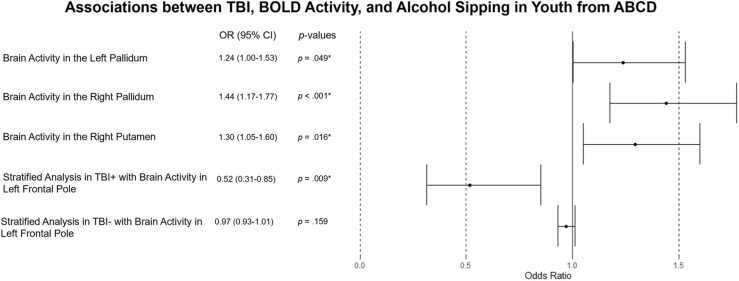
Moderated Logistic Regression Results.

Using the Generalized Estimating Equation (GEE Pack) (Halekoh et al., 2006, Yan and Fine, 2004, Yan, 2002) package for R Studio, multilevel moderated logistic regression analyses were performed controlling for household income (continuous), biological sex, UPPS-P Positive Urgency (continuous; see Fig. 1), and age (continuous). The main effect of TBI was not found to be a significant predictor of alcohol sipping in any model (*p*’s > .100). However, results demonstrated significant main effects of EN-Back BOLD activity in both the left (OR = 1.238, 95% CI [1.001–1.531], *p* = .049) and right (OR = 1.442, 95% CI [1.174–1.770], *p* < .001) pallidum, where increased BOLD activity at year 2 was associated with increased odds of having sipped alcohol at year two, controlling for TBI and the other covariates. Increased BOLD activity in the left pallidum was associated with a 24% increased likelihood of having sipped alcohol and for increased BOLD activity in the right pallidum a 44% increased likelihood of having sipped alcohol. A significant main effect of BOLD activity in the right putamen was also found (OR = 1.295, 95% CI [1.050–1.598], *p* = .016), controlling for TBI and covariates, where increased BOLD activity at year 2 was associated with increased odds of having sipped alcohol at year two. Increased BOLD activity in the right putamen was associated with a 30% increased likelihood of having sipped alcohol.

#### 2.1.2 Interaction effects

**Fig. 2:**
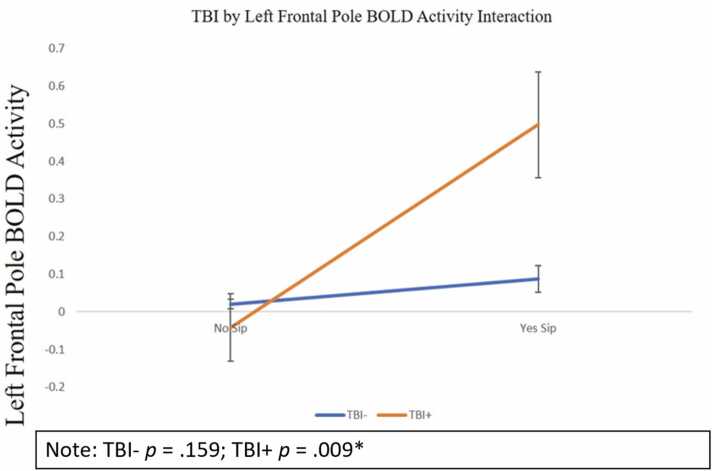
Interaction Plot.

Although there were not significant main effects of BOLD activity in the left frontal pole (OR = 0.971, 95% CI [0.932 −1.012], *p* = .163) or TBI (OR = 1.070, 95% CI [0.625 −1.831], *p* = .702805 there was a significant interaction between TBI and BOLD activity in the left frontal pole on odds of alcohol sipping (OR = 0.507, 95% CI [0.303 – 0.846], *p* = .009). To interpret the interaction, subsequent simple slope analyses were performed stratified by TBI-status. These analyses were simple logistic regression analyses examining if BOLD activity in the left frontal pole could predict odds of alcohol sipping for (1) those who are TBI-negative and (2) those who are TBI-positive. For individuals who did not report experiencing a TBI at baseline there was no significant association between BOLD activity in the left frontal pole and alcohol sipping (OR = 0.971, 95% CI [0.931 −1.012], *p* = .159). However, for individuals who experienced a TBI there was a significant association between BOLD activity in the left frontal pole and odds of having sipped alcohol, whereby increased BOLD activity in the frontal pole was associated with decreased odds of having sipped alcohol (OR = 0.516, 95% CI [0.314 – 0.850], *p* = .009; see Fig. 2). This indicates that for those who have experienced a TBI, increased BOLD activity in the left frontal pole was associated with a 48% decreased likelihood of having sipped alcohol. Analyses were limited to 10 a priori regions of interest for this analysis, however, a false discovery rate procedure was applied for a multiple test adjustment on all main effects, interactions, and simple slope analyses. After FDR adjustment, only the main effect of BOLD activity in the right pallidum survived correction.

## 3 Discussion

In the present study we hypothesized that: (1) the presence of a TBI would predict increased odds of alcohol experimentation, (2) that increased BOLD activity in the fronto-basal ganglia network during the EN-Back task would predict increased odds of alcohol experimentation, and (3) that TBI and BOLD activity would interact to predict odds of alcohol experimentation. The first hypothesis was not supported as no significant main effect of TBI was identified. The lack of findings with regards to TBI predicting alcohol experimentation may be related to low prevalence rates of TBI with loss of consciousness (i.e., 0.01%) in the ABCD cohort to date. Although mild TBI (2.71%) had higher prevalence rates than TBI with loss of consciousness, this study chose to examine the impact of more severe TBIs that led to an altered cognitive state (i.e., loss of consciousness). The second hypothesis was supported, where increased levels of BOLD activity during the EN-Back task was associated with increased odds of alcohol experimentation in both the left and right pallidum alongside the right putamen. Finally, the third hypothesis was partially supported by an interaction between TBI-status and left frontal pole BOLD activity on odds of alcohol experimentation. This interaction demonstrated that increased BOLD activity (a proxy measure of increased levels of neuronal activity) under working load was significantly associated with decreased odds of alcohol experimentation, only for those reporting mild and/or severe TBI by ages 9–10.

TBIs have been found to be associated with alcohol misuse in previous studies (Bogner et al., 2019), yet no studies to-date have examined this in children as young as 9–10 years-old. For hypothesis one, it was unexpected that no significant main effect of TBI was identified, which may simply be related to the low prevalence of TBIs and alcohol experimentation in this sample. This effect may become more pronounced as the children develop and begin having full drinks, as opposed to just sipping. With regards to hypothesis two we identified the bilateral pallidum and the right putamen as regions where increased activation was associated with increased odds of alcohol sipping. This activation pattern was as expected for the basal ganglia regions (Aron et al., 2007; Lize et al., 2017) and frontal region (Olsen et al., 2014). The left and right pallidum were the only anatomical regions identified bilaterally, which may indicate that the activity in the pallidum is especially relevant to the initiation of alcohol use amongst this sample of ABCD study youth. The association between the right pallidum and alcohol experimentation was found to be stronger than the left pallidum, comparing an increased likelihood of 44% to an increased likelihood of 24%. The pallidum is of particular relevance as it is tied to reward and motivation behaviors as part of the limbic-reward pathway (Smith et al., 2009). The findings comprising the putamen and pallidum were of particular interest as these regions are part of the fronto-basal ganglia network, which is implicated in reward-associated learning and motivation, inhibitory control (Aron et al., 2007), and aspects of attention and working memory (Hermans et al., 2017, Olsen et al., 2015). These functions have implications for better understanding reward-seeking behavior related to substance misuse.

As for the interaction for hypothesis three, we found that those in the TBI-positive group demonstrated increased activation in the left frontal pole that was associated with decreased odds of having sipped alcohol. It was initially expected that increased activity in frontal regions would be associated with odds of having sipped alcohol due to the increased demand of cognitive load post injury. Yet, increased activity in the left frontal pole was predictive of decreased odds of sipping, and this association may indicate a divergent neuromaturational profile, or neuroprotection, such that increased activity in this region following a TBI (or in those predisposed to TBI-associated behaviors and traits) may show divergent neurovascular responses over the course of development that is not necessarily indicative of pathology but advantageous. (Harris et al., 2011). This difference in BOLD activity could be an indicator that in this subset of individuals and/or post-TBI recovery, greater neural activity may be related to increased inhibitory control and reduced likelihood of alcohol usage. While BOLD is a reflection of neuronal activity with relatively high spatial resolution (Hall et al., 2016), additional studies are needed for understanding how changing brain metrics over time relate to TBI and alcohol misuse (Harris et al., 2011).

## 4 Limitations and directions for future research

Although novel, this study was subject to several limitations. First, there was a low prevalence of TBI (0.01%, 134/11,876) and alcohol experimentation (11%, 1184/11,876) rates in the sample, which was not surprising given that the participants were young (ages 9–13). Second, the present study did not include an equal quantity of mild, moderate, and severe TBI given the small number of TBI cases in the cohort to-date. It is also possible that time between TBIs, time since first TBI, and time since most recent TBI are important factors in behavioral outcomes, and future work from our laboratory will explore these factors as TBI rates are likely to increase in the cohort as the youth get older. Additionally, we did not examine the specific number of sips as a continuous variable, as we were primarily interested in examining experimentation and early initiation of alcohol use as opposed to the quantity of use. Impulsivity was also used as a covariate in our analyses, but it is possible that it may be a mediator variable of the association between TBI and alcohol experimentation.

Multiple imputation was conducted to reduce bias in the sample (Van Buuren and Groothuis-Oudshoorn, 2011); however, missing fMRI data in the sample may result in less precise statistical estimates. Although there were some differences in missing data based on highest household income, this variable was controlled for as a covariate. Importantly, there was no difference in missingness by TBI group or sipping group status. Given the substantial number of planned analyses (i.e., 20 moderated logistic regression analyses) we applied a multiple comparison correction and several findings did not survive false discovery rate correction. Additionally, large sample studies such as ABCD provide sufficient statistical power to detect rather small effect sizes that are statistically significant, and thus replication and minimization of measurement error will be important for ongoing examination of the associations between TBI and substance use outcomes in the ABCD Study cohort. Nevertheless, the findings from the present study may still have clinically-relevant implications and provide direction for future studies designed to examine neural health, head injury, and substance misuse (Owens et al., 2021). Our laboratory will continue to examine these associations with more time points and variability in TBI and alcohol use status. With regards to generality of the study’s findings, it is also important to acknowledge the limitations of the data. Although the sample was recruited to be broadly representative of the demography of the United States (Garavan et al., 2018), individuals who self-select to participate in longitudinal research are often families of higher SES and educational levels (Compton et al., 2019). Lastly, the study only examined BOLD activity under working memory cognitive load, whereas resting state BOLD or brain structure estimates were beyond the scope of this present study.

Despite the implications of this study, there is still much room for research in this area. Although used as a covariate, future studies should examine in detail the impact that personality factors such as impulsivity have on the association between TBI and alcohol experimentation in youth as a potential moderator. Impulsivity in ABCD has been identified as a risk factor for both alcohol curiosity (Wade et al., 2021) alongside alcohol sipping (Watts et al., 2021). Impulsivity has also been found to be a significant mediator of the association between TBI before age 12 and conduct disorder symptoms at age 16 (Khalaf et al., 2023), yet the interaction between TBI and impulsivity on alcohol experimentation behaviors in adolescents has yet to be examined. Future studies should further investigate the potential mediating role of impulsivity on the association between TBI and alcohol use in ABCD alongside the potential for a three-way interaction between TBI, BOLD activity in the fronto-basal ganglia network, and impulsivity on alcohol use. Although this study identified brain ROIs where BOLD activity was associated with alcohol experimentation, it did not examine structural integrity (e.g., gray matter volumes, white matter tissue health). Given the findings of the present study and past literature identifying reduced gray matter volumes in regions of the frontal lobe following heavy alcohol consumption in adolescence (Heikkinen et al., 2017; Tapert and Eberson-Shumate, 2022), structural imaging metrics should also be further examined in youth from the ABCD cohort who have experienced a TBI. Given the bilateral findings surrounding the pallidum in this study, future studies should examine if this difference is due to a functional difference in the brain or if this is also related to macrostructural differences in tissue architecture. Finally, the association between TBI, BOLD activity, and alcohol use should be examined across a larger period of time to better understand the long-term impact of a TBI in childhood and adolescence including examination of multiple TBIs and substance misuse characteristics using future ABCD timepoints.

## 5 Conclusion

In summary, adolescent TBI and EN-Back-related BOLD activity in areas of the fronto-basal ganglia were found to be associated with increased odds of alcohol experimentation two-years after TBI and functional brain imaging assessment, at ages 11–13. Decreased BOLD activity in the frontal pole was associated with increased odds of alcohol experimentation in the TBI-positive group only. Future prospective studies on the association between TBI, frontal-basal ganglia functional brain activity, and alcohol misuse should be conducted to better understand the complex relationships between these constructs over the course of development to inform age-appropriate interventions for TBI during adolescence.

## Funding

Research was supported by the 10.13039/100000026National Institute on Drug Abuse grants U01 DA041089, R21 DA047953, R01 DA054106, R01 DA054980, and the California Tobacco-Related Disease Research Grants Program Office of the University of California grants 580264 and T30IP0962 (Dr. Joanna Jacobus). This work was also supported in part by a National Institute of Child Health and Human Development (10.13039/100000071NICHD) grant R01 HD105338 (Dr. Jeffrey Max), by the National Institute on Alcohol Abuse and Alcoholism (10.13039/100000027NIAAA) grant T32 AA013525 (Everett Delfel), the National Institute of Mental Health (10.13039/100000025NIMH) grant T32 MH018399 (Dr. Kelly Correa), and the National Center for Advancing Translational Sciences (10.13039/100006108NCATS) grant UL1TR001442 and KL2TR001444 (Dr. Laika Aguinaldo). The content is solely the responsibility of the authors and does not necessarily represent the official views of the 10.13039/100000002NIH.

## CRediT authorship contribution statement

**Tapert Susan:** Writing – review & editing, Supervision, Project administration, Methodology, Investigation, Funding acquisition, Conceptualization. **Jacobus Joanna:** Writing – review & editing, Writing – original draft, Visualization, Validation, Supervision, Project administration, Methodology, Investigation, Funding acquisition, Conceptualization. **Delfel Everett Lee:** Writing – original draft, Visualization, Validation, Methodology, Investigation, Formal analysis, Data curation, Conceptualization. **Aguinaldo Laika:** Writing – review & editing, Visualization, Supervision, Methodology, Formal analysis, Data curation, Conceptualization. **Courtney Kelly E.:** Writing – review & editing, Visualization, Supervision, Methodology, Conceptualization. **Max Jeffrey E.:** Writing – review & editing, Supervision, Methodology, Conceptualization. **Correa Kelly:** Writing – review & editing, Methodology, Formal analysis, Data curation.

## Declaration of Competing Interest

The authors declare that the research was conducted in the absence of any commercial or financial relationship that could be construed as a potential conflict of interest. Dr. Max provides expert testimony in cases of traumatic brain injury on an ad hoc basis for plaintiffs and defendants on a more or less equal ratio. This activity constitutes approximately 5% of his professional activities.

## Data Availability

The authors do not have permission to share data.
